# Family's presence in the pediatric emergency room: opinion of health's professionals

**DOI:** 10.1016/j.rpped.2015.03.010

**Published:** 2015

**Authors:** Francine Fernandes Pires Mekitarian, Margareth Angelo

**Affiliations:** a Hospital Universitário da Universidade de São Paulo (USP), São Paulo, SP, Brazil; b Universidade de São Paulo (USP), São Paulo, SP, Brazil

**Keywords:** Pediatrics, Emergency medical services, Patient care team, Professional-family relations

## Abstract

**Objective:**

To learn the opinion of health professionals regarding the presence of family during pediatric emergency care.

**Methods:**

Cross-sectional study, performed with 46 health professionals, members of the medical and nursing team of a pediatric emergency service. The data were collected via the application of a questionnaire composed by variables related to the opinion of professionals about the studied subject, in line with the professional category and the vocational training time, as well as invasive procedures during which the presence of family is authorized by the professionals.

**Results:**

The medical staff and the professionals with shorter time after graduation (<10 years) were more favorable to the presence of family during emergency procedures. Regarding the complexity of the procedures, the nursing staff proved more favorable to the presence of family during less complex procedures - peripheral venous puncture and fluid sample - whereas the consent of the medical staff was similar, regardless the performed procedure - peripheral venous puncture, fluid sample, intraosseous puncture, tracheal intubation and cardiopulmonary resuscitation.

**Conclusions:**

In order to allow the presence of family in the emergency room, it is necessary to sensitize health professionals, especially the nursing staff and the longer-term acting professionals, which are more resistant to allow the family to stay with the child during the emergency care.

## Introduction

Family-Centered Care (FCC) is a global trend in health care provision. Its main premise is that the assistance shall be planned for the entire family and not just for the patient. This care model started between the decades of 1950-60, from the moment when the negative psychological effects that physical separation of parents had on children were recognized, and therefore parents could be included in the care of their child within the hospital environment.^1^

In Brazil, this model of care is not yet integrated into the health care services. Although the Child and Adolescent Statute (Estatuto da Criança e do Adolescente) recommends the permanent presence of parents next to the hospitalized child, it is not only this presence that will ensure that FCC will be followed.^2^ In addition to allowing the presence of the family, one must also recognize the family needs, perspectives and choices.^3^

According to the FCC guideline, families should be encouraged to participate in patient care at all levels of hospital care.^3^Considering the introduction of this model of care in the emergency room, it becomes necessary to allow families to decide whether they want or not to be close to the patient during invasive and cardiopulmonary resuscitation (CPR) procedures.^4^

Although international organizations, such as the American Heart Association and the Emergency Nurses Association, recommend a model of care that favors the presence of family members during invasive and CPR procedures in the emergency room, family members are still invited by health professionals to leave the room.^5^^,^ 6 It is essential that such practice be normalized by institutional protocols, so that this decision does not depend exclusively on the healthcare team.^4^

In general, as it is the healthcare team who decides whether the family can stay in the emergency room, it is important to understand their perspectives. Health professionals justify that they ask the families to leave for fear the family will lose emotional control and interfere with the provided care. They also believe that, with the family present during care, the professionals might feel anxious, especially the professionals in training, which could interfere with their capacity and concentration when performing the procedures. Additionally, they report the fear that the family will keep stressful memories related to the care, even more so in unfavorable outcomes.^7^ Although there is no scientific evidence to support these beliefs, this remains a widely debatable subject.

Even though there are international publications on the subject, there are no national studies that have specifically addressed this issue, which justifies the need to know the opinions of health professionals working in pediatric emergency services in our cultural scenario. Thus, this study aims to know the opinions of health care professionals working in a Brazilian child emergency service in relation to the presence of family members in the room during emergency care. The outcomes related to the professional's consent or not for the presence of family members in the pediatric emergency room were: professional's previous experience with family in that environment; professional category; time since graduation and type of invasive procedure performed.

## Method

This was a cross-sectional study, performed by applying a questionnaire in the child emergency room of Hospital Universitário (HU) of Universidade de São Paulo (USP). The study site is a secondary-care public teaching hospital, which treats children from birth to 14 years of age. This service has no protocol that regulates the presence of family members in the emergency room, but this practice is often accepted by health professionals in the unit. The decision to allow or not the presence of family members in the emergency room is made at the time of treatment.

Study subjects were professionals from the multidisciplinary team working in the emergency care service, consisting of physicians and medical residents, nurses, nursing technicians and assistants. The other professionals of the multidisciplinary team were not included, because only the medical and nursing staffs work at the study site. No exclusion criteria were defined to select the sample.

The participants were addressed by the main investigator, a nurse from that sector, and received, after signing the Informed Consent Form, a questionnaire consisting of variables related to age, gender, professional category, time since graduation, whether professionals had had a previous experience with the presence of family members in the emergency room (yes or no) and during which invasive procedures health professionals would allow the presence of the family (peripheral venous puncture, CSF sample collection, intraosseous puncture, intubation and CPR). For this last item, the participants were able to check as many items as they considered adequate.

To better understand the views of professionals on the assessed subject, qualitative questions were included, with the first one being "who do you think must decide whether the family can stay in the emergency care room?" Another question was also made that allowed assessing the reasons why professionals include or exclude the families from the room - "What do you think about the presence of family members in the emergency room during the child's care?"

The questionnaire was created by the authors, based on evidence of studies available in the international literature. The final version was submitted to examiners - professionals that were experts in the assessed subject and experienced in the construction of tools - to assess the clarity and the semantic aspect of the questions. Considering the positive tool assessment, a pilot study was not carried out. The participants agreed to return the filled out questionnaire to the main investigator one week after receiving it.

To allow statistical analysis, the professionals were grouped according to their professional category: medical staff (doctors and residents) and nursing staff (nurses, nursing technicians and assistants). Another group was created in relation to time since graduation: less than 10 years and 10 years or more after graduation. As the professionals’ answers to the qualitative question "who do you think should decide whether the family can stay in the emergency care room?" were only: family or health professionals, these two groups of responses were grouped and the statistical analysis steps were followed, according to the variables professional category and time since graduation.

In order to verify the existence of a statistical association between allowing the presence of family members in the emergency room, the professional category, time since graduation and who must decide whether the family can be present during care, chi-square test or Fisher's exact test were used.^8^ Regarding the association between allowing the presence of family members in the emergency room and the type of procedure performed, generalized estimating equations with binomial distribution function and logit association were applied.^9^ All tests were performed using a 5% significance level, and SPSS15.0 software (IBM, Chicago, USA) was used for the statistical analyses.

The analysis of the answers given to the qualitative question "What do you think about the presence of family members in the emergency room during the child's care?" followed the thematic coding steps.^10^Initially, the data were interpreted individually and codes were developed that were appropriate to the opinion of each participant. Subsequently, the data were grouped by their similarities, among them the reasons why professionals allow or do not allow families to stay in the emergency room.

The study was approved by the HU USP's (protocol 1182/12) and Escola de Enfermagem da USP's Institutional Review Board (protocol 1102/2011). The data collection was carried out in March 2012.

## Results

All health unit professionals (*n*=46) were invited to participate in the study, and the return rate of the questionnaires was 100%. Most professionals were females (78.3%) and belonged to the nursing staff (56.5%), reported they had already provided care in the emergency room when the family was present (95.7%), and had 10 years or more since graduation (65.2%). When considering the distribution among the professional categories and the time since graduation: 70% of the nursing team had 10 or more years since graduation, while only 30% of the medical staff had the same period of time since graduation.

As almost the entire sample had had the experience of providing care with the presence of the family in the emergency room, the statistical association between this variable and the professionals’ opinion regarding the analyzed practice was not carried out.

According to Table 1, the opinion of the professionals regarding the presence of family members in the emergency room was not associated to the professional category for any of the studied procedures. The data suggest, however, that the medical staff was more favorable to the presence of the family for all assessed invasive procedures, compared to the nursing staff.

**Table 1:** Health care professionals’ opinion regarding the presence of family members in the pediatric emergency room, according to the professional category and type of procedure performed.

Invasive procedure/favorable to the family's presence?	Medical staff	Nursing staff	Total	*p*-value
	*n* (%)	*n* (%)	*n* (%)	
*Peripheral venous puncture*				
Yes	20 (100.0)	26 (100.0)	46 (100.0)	a

*CSF collection*				
Yes	19 (95.0)	23 (88.5)	42 (91.3)	0.622
No	1 (5.0)	3 (11.5)	4 (8.7)	

*Intraosseous puncture*				
Yes	15 (75.0)	15 (57.7)	30 (65.2)	0.222
No	5 (25.0)	11 (42.3)	16 (34.8)	

*Tracheal intubation*				
Yes	14 (70.0)	11 (42.3)	25 (54.3)	0.062
No	6 (30.0)	15 (57.7)	21 (45.7)	

*CPR*				
Yes	13 (65.0)	11 (42.3)	24 (52.2)	0.127
No	7 (35.0)	15 (57.7)	22 (47.8)	

*Total*	20 (100.0)	26 (100.0)	46 (100.0)	

CSF, cerebrospinal fluid; CPR, cardiopulmonary resuscitation.

a All professionals accept the family's presence for this procedure.

Table 2 shows that professionals with less time since graduation considered, as compared to those with 10 years or more since graduation, that the family can be present while the staff is performing tracheal intubations and CPR procedures (*p*=0.007 and *p*=0.024, respectively). For other procedures, the data also indicate, although without statistical association, that professionals more recently graduated are more favorable to the family's presence in the emergency room.

**Table 2:** Health care professionals’ opinion regarding the family's presence in pediatric emergency room, according to time since graduation and type of procedure.

Invasive procedure/favorable to the family's presence?	Time since graduation		Total	*p*-value
	<10 years *n* (%)	≥10 years *n* (%)	*n* (%)	
*Peripheral venous puncture*				
Yes	16 (100.0)	30 (100.0)	46 (100.0)	a

*CSF collection*				
Yes	16 (100.0)	26 (86.7)	42 (91.3)	0.282
No	0 (0.0)	4 (13.3)	4 (8.7)	*

*Intraosseous puncture*				
Yes	13 (81.3)	17 (56.7)	30 (65.2)	0.095
No	3 (18.8)	13 (43.3)	16 (34.8)	

*Tracheal intubation*				
Yes	13 (81.3)	12 (40.0)	25 (54.3)	0.007
No	3 (18.8)	18 (60.0)	21 (45.7)	

*CPR*				
Yes	12 (75.0)	12 (40.0)	24 (52.2)	0.024
No	4 (25.0)	18 (60.0)	22 (47.8)	

*Total*	16 (100.0)	30 (100.0)	46 (100.0)	

CPR, cardiopulmonary resuscitation.

a All professionals accept the family's presence for this procedure.

Most professionals (69.8%) believe that it is the health professionals’ decision to allow or not families to stay during treatment in the emergency room. Professionals with less than 10 years since graduation are more inclined to accept (*p*=0.034) that families should participate in this decision (Table 3).

**Table 3:** Opinion about who should decide if the family may or may not be present during emergency procedures in a pediatric emergency room, according to professional category and time since graduation.

	Who should decide whether or not the family can stay in the emergency room		*p*-value
	Family *n* (%)	Health care professionals *n* (%)	
*Professional category*			0.707
Medical staff	6 (33.3)	12 (66.7)	
Nursing staff	7 (28.0)	18 (72.0)	

*Time since graduation*			0.034
<10 years	(53.3)	7 (46.7)	
≥10 years	5 (17.9)	23 (82.1)	

*Total*	13 (30.2)	30 (69.8)	

Three cases were excluded from this analysis due to the impossibility of determining the answer to this question.

Regarding the association between the presence of family members in the pediatric emergency room and the complexity of the performed procedures, the following increasing order of acceptance was observed (Table 4): peripheral venous puncture, CSF sample collection, intraosseous puncture, tracheal intubation and CPR. The nursing staff was more favorable to the family's presence in less complex procedures (peripheral venous puncture and CSF sample collection; *p*<0.001) compared to more complex procedures (intraosseous puncture, intubation and CPR). However, the agreement of the medical team regarding the presence of family members was similar (*p*=0.124), regardless of the performed procedure.

**Table 4:** Opinion of each professional category regarding the presence of family members in the pediatric emergency room according to the procedure performed.

Professional category	Invasive procedure	Favorable to the presence of family members in the emergency room?		Total	*p*-value
		No *n* (%)	Yes *n* (%)		
Nursing staff	Peripheral venous puncture	0 (0.0)	26 (100.0)	26	<0.001
	CSF collection	3 (11.5)	23 (88.5)	26	
	Intraosseous puncture	11 (42.3)	15 (57.7)	26	
	Tracheal intubation	15 (57.7)	11 (42.3)	26	
	CPR	15 (57.7)	11 (42.3)	26	

Medical staff	Peripheral venous puncture	0 (0.0)	20 (100.0)	20	0.124
	CSF collection	1 (5.0)	19 (95.0)	20	
	Intraosseous puncture	5 (25.0)	15 (75.0)	20	
	Tracheal intubation	6 (30.0)	14 (70.0)	20	
	CPR	7 (35.0)	13 (65.0)	20	

Total	Peripheral venous puncture	0 (0.0)	46 (100.0)	46	<0.001
	CSF collection	4 (8.7)	42 (91.3)	46	
	Intraosseous puncture	16 (34.8)	30 (65.2)	46	
	Tracheal intubation	21 (45.7)	25 (54.3)	46	
	CPR	22 (47.8)	24 (52.2)	46	

The reasons why professionals include families in the emergency room were: to have the family observe their efforts to save the child's life; to have the family provide important information about the patient; it is the family's right to be there, and to have the family provide reassurance to the child. The reasons that led them to exclude families were: professionals do not have time to pay attention to the families, the family presence hinders training provided to students, the family interferes with the professionals’ work, the need for a professional to stay with the family, and family keeps negative memories of the care.

## Discussion

This study is a pioneer in the national literature on the opinion of health professionals working in a children's emergency room regarding the presence of family members in the ER. As, in general, the health professionals are the ones who decide whether the family will be allowed to stay in the emergency care room, knowing their opinions is essential to draw strategies that can modify the health care team's approach and recognize the family's needs in the emergency environment.

The number of professionals who reported having participated in an emergency call when the family was present is far superior to those previously reported in the literature - 23.1-70.1%.^11^^,^ 12 This finding could mean a positive scenario for the implementation of strategies of the FCC care model, as the previous experience with the presence of family members in the emergency room has shown to be the most favorable determinant for attaining this practice.^13^

Although the medical staff was more favorable to the presence of family members in the emergency room than the nursing staff, this result contrasts those described in the literature.^14^^,^ 15 It is noteworthy that the studies described in the international literature do not include the perspectives of nursing assistants and technicians.

The fact that professionals with less time since graduation are more favorable to the presence of family members during emergency care reinforces the observed results regarding the fact that the medical staff was more favorable to the family's presence in the emergency room, since most of these professionals (70%) had graduated less than 10 years before. There is no correlation in the literature between time of experience in emergency services and actions taken by the professionals regarding the presence of the family in the emergency room.^16^

Most professionals believe that the family should not participate in the decision-making regarding their own presence in the emergency room. These data mainly reflect the views of the professionals with longer time since graduation. Results available in the literature also show that health professionals disregard the family's autonomy in this decision-making process.^12^^,^ 17

The increased practice related to less invasive procedures in daily life seems to give professionals a greater degree of confidence in carrying out these procedures, which could explain the findings in the literature,^17^^,^ 18 which show that the more invasive the procedure, the less often professionals are favorable to the presence of the family. However, in our study, only the nursing staff demonstrated this preference regarding the family presence only for the less invasive procedures.

The reasons that led professionals to allow or not the presence of family members in the emergency room were similar to those given by doctors and nurses in international studies, showing that the cultural context had little impact on the perception of the health team to decide to allow or not the family to be present during emergency care.^19^^-^ 21 It is essential that the needs of the families, even in emergency situations, be taken into consideration during the provided care, as the family's presence in a situation in which the health team members are not prepared to recognize their needs may have more negative consequences for the families than not being close to their relative.^22^ Studies have shown that, when the option is given, the families usually choose to stay during emergency care, and their presence helps the mourning process in case of death, provides a more effective communication with the multidisciplinary team and allows them to verify that all the necessary efforts were made to save the patient's life.^23^^,^ 24

A favorable environment to promote the family's presence in the emergency room includes the development of a professional awareness program for the team's capacitation to include families in this scenario and recognize the benefits that the family members bring to emergency care.^25^It is necessary to create strategies to allow families to be present in the emergency room, by considering their needs. This can be achieved by assigning a health care team member to stay exclusively with the family, providing support to their psychosocial needs, which may contribute to the lack of stressful memories in the future. In addition, this professional - which can be any member of the multidisciplinary team - must evaluate the family's emotional responses to the procedures, anticipating possible interruptions during care.^26^ However, to create this favorable context in our cultural reality, first of all it is necessary to recognize that the Brazilian pediatric emergency situation differs from those described in the international scenario, and it is marked by health professionals’ work overload due to the overcrowding of emergency rooms and by limited resources - human, physical, and available materials. Moreover, unrestricted access to emergency services results in long waiting time for users, which causes distress and stress to the health team, who performs under a greater work demand than they are prepared to meet.^27^^,^ 28

This study has as limitation the exposure of professionals’ perception in a given reality and context that cannot be fully replicated in other emergency scenarios. However, it is an excellent tool for future studies to establish strategies to include families in the emergency room. It is still necessary to carry out national studies that assess the perceptions of families in this context and the creation of a protocol that will regulate this care practice, adapted to our care and cultural reality.

Finally, the study concludes that the medical staff and health care professionals with less than 10 years since graduation are more favorable to the presence of family members in the emergency room when compared to the other professionals. The reasons why professionals do not allow family members to stay in the emergency room are related to discomfort and insecurity felt by professionals due to the presence of family members during care. On the other hand, the reasons that make them allow the presence of the families in this scenario are often associated to the objective of facilitating their own work, disregarding the families’ real needs.
