# Supplementary material for: Family's presence in the pediatric emergency room: opinion of health's professionals
Source: Rev Paul Pediatr. 2015 Oct-Dec;33(4):460–6. doi: 10.1016/j.rpped.2015.03.010 (PMC4685567; doi:10.1016/j.rpped.2015.03.010)
Supplement: Translation

# Translation: Presença da família em sala de emergência pediátrica: opiniões dos profissionais de saúde

**Authors:** Francine Fernandes Pires Mekitarian, Margareth Angelo

**Affiliations:** a Hospital Universitário da Universidade de São Paulo (USP), São Paulo, SP, Brasil; b Universidade de São Paulo (USP), São Paulo, SP, Brasil

**Keywords:** Pediatria, Serviços médicos de emergência, Equipe de assistência ao paciente, Relações profissional-família

## Abstract

**Objetivo:**

Conhecer as opiniões de profissionais de saúde em relação à presença da família durante o atendimento em sala de emergência pediátrica.

**Métodos:**

Estudo transversal, feito com 46 profissionais de saúde, integrantes das equipes médica e de enfermagem de um serviço de pronto-socorro infantil. Os dados foram coletados por meio da aplicação de um questionário composto por variáveis relacionadas à opinião dos profissionais em relação ao tema estudado, associada à categoria profissional e ao tempo de formação profissional, além de quais procedimentos invasivos em que os profissionais autorizam a presença da família.

**Resultados:**

A equipe médica e os profissionais com menor tempo de formação (<10 anos) foram mais favoráveis à presença da família durante os procedimentos de emergência. Em relação à complexidade dos procedimentos, a equipe de enfermagem se mostrou mais favorável à presença da família em procedimentos menos complexos - punção venosa periférica e coleta de líquor -, enquanto a concordância da equipe médica foi similar, independentemente do procedimento feito - punção venosa periférica, coleta de líquor, punção intraóssea, intubação traqueal e reanimação cardiopulmonar.

**Conclusões:**

Para permitir a presença da família em sala de emergência, é necessário sensibilizar profissionais de saúde, especialmente a equipe de enfermagem e os profissionais formados há mais tempo, que são mais resistentes a permitir que a família fique ao lado da criança durante o atendimento de emergência.

## Introdução

O Cuidado Centrado na Família (CCF) é uma tendência mundial para se prestarem os cuidados em saúde. Sua principal premissa é que a assistência seja planejada para toda a família, e não somente para o indivíduo doente. Esse modelo de cuidado iniciou-se entre as décadas de 1950-60, a partir do momento em que foram reconhecidos os efeitos psicológicos negativos que a separação física dos pais causava nas crianças e, assim, os pais puderam ser incluídos no cuidado ao filho, dentro do ambiente hospitalar.^1^

No Brasil, esse modelo de cuidado ainda não está incorporado nos serviços de saúde. Embora o Estatuto da Criança e do Adolescente recomende a permanência integral dos pais ao lado da criança hospitalizada, não é somente essa presença que garantirá que o CCF seja feito.^2^ Além de permitir a presença familiar, é preciso ainda reconhecer as necessidades, perspectivas e escolhas das famílias.^3^

Como diretriz do CCF, as famílias devem ser encorajadas a participar da assistência ao paciente em todos os níveis do cuidado hospitalar.^3^ Pensando-se na introdução desse modelo de cuidado no ambiente de emergência, torna-se necessário permitir que as famílias decidam se gostariam de estar ou não ao lado do paciente durante procedimentos invasivos e de reanimação cardiopulmonar (RCP).^4^

Embora organizações internacionais, como a American Heart Association e a Emergency Nurses Association, recomendem uma prática assistencial favorável à presença da família durante procedimentos invasivos e RCP na sala de emergência, os parentes ainda são convidados pelos profissionais de saúde a se retirar do atendimento.^5^^,^ 6 É fundamental que essa prática seja normatizada por protocolos institucionais, para que essa decisão não dependa exclusivamente da equipe de saúde.^4^

Como, em geral, são os profissionais de saúde que decidem se a família poderá estar presente na sala de emergência, é importante compreender suas perspectivas. Os profissionais de saúde justificam que excluem a família desse cenário por medo de que perca o controle emocional e interfira no cuidado prestado. Acreditam ainda que com a família presente durante o atendimento, os profissionais sentir-se-iam ansiosos, especialmente os profissionais em treinamento, o que poderia interferir em sua habilidade e concentração na feitura dos procedimentos. Além disso, relatam o receio de que a família permaneça com memórias estressantes relacionadas ao atendimento, ainda mais nos desfechos desfavoráveis.^7^ Embora, não existam evidências científicas que respaldem tais crenças, esse ainda é um tema de ampla discussão.

Embora existam publicações internacionais sobre a temática, não há estudos nacionais que abordem especificamente esse assunto, o que justifica a necessidade de conhecer as opiniões dos profissionais de saúde que atuam em serviços de emergência pediátricos em nosso cenário cultural. Sendo assim, este estudo tem como objetivo conhecer as opiniões de profissionais de saúde que atuam em um serviço de pronto-socorro infantil brasileiro em relação à presença da família durante o atendimento de sala de emergência. Os desfechos associados ao fato de o profissional permitir ou não a presença da família na sala de emergência pediátrica foram: experiência prévia do profissional com a família nesse ambiente; categoria profissional; tempo de formação profissional e tipo de procedimento invasivo.

## Método

Estudo transversal, feito por meio da aplicação de questionário no pronto-socorro infantil do Hospital Universitário (HU) da Universidade de São Paulo (USP). O local de estudo é um hospital escola público, de nível secundário, que atende crianças desde o nascimento até 14 anos. Esse serviço não tem protocolo que regulamenta a presença da família em sala de emergência, mas essa é uma prática adotada, com certa frequência, pelos profissionais de saúde da unidade. A decisão sobre permitir ou não a presença da família na sala de emergência é tomada do momento do atendimento.

Foram sujeitos do estudo os profissionais da equipe multidisciplinar que atua nos atendimentos de emergência do serviço, composta por médicos assistentes e residentes, enfermeiros, técnicos e auxiliares de enfermagem. Não foram incluídos outros profissionais da equipe multiprofissional, pois somente a equipe médica e de enfermagem atua no local do estudo. Não foram definidos critérios de exclusão para selecionar a amostra.

Os participantes foram abordados pela pesquisadora principal, que é enfermeira do referido setor, e receberam, após assinar o termo de Consentimento Livre e Esclarecido, um questionário composto por variáveis relacionadas a: idade, sexo, categoria profissional, tempo de formação profissional, se os profissionais já tiveram uma experiência prévia com a família em sala de emergência (sim ou não) e em quais procedimentos invasivos os profissionais de saúde permitiriam a presença da família (punção venosa periférica, coleta de líquor, punção intraóssea, intubação traqueal e RCP). Para esse último item, os participantes puderam assinalar quantos itens julgassem necessário.

Para compreender melhor a opinião dos profissionais em relação ao tema estudado, foram incluídas questões qualitativas: a primeira, "quem você considera que deve decidir se a família pode permanecer no atendimento de emergência?". Foi apresentada ainda outra questão que permitiu acessar os motivos pelos quais os profissionais incluem ou excluem as famílias do atendimento - "O que você pensa sobre a família permanecer durante o atendimento da criança na sala de emergência?"

O questionário foi construído pelas autoras, com base nas evidências dos estudos disponíveis na literatura internacional. A versão construída foi submetida a juízes - profissionais peritos no tema estudado e experientes na construção de instrumentos - para avaliação da clareza e do aspecto semântico das perguntas. Considerando a avaliação positiva do instrumento, não foi feito estudo-piloto. Os participantes concordaram em devolver o questionário preenchido à pesquisadora principal uma semana após sua entrega.

Para permitir a análise estatística, os profissionais foram agrupados segundo sua categoria profissional em: equipe médica (médicos assistentes e residentes) e equipe de enfermagem (enfermeiros, técnicos e auxiliares de enfermagem). Outro agrupamento foi em relação ao tempo de formação profissional: menos de 10 anos e 10 anos ou mais de formado. Como as respostas que os profissionais deram à questão qualitativa "quem você considera que deve decidir se a família pode permanecer no atendimento de emergência?" foram somente família ou profissionais de saúde, esses dois grupos de respostas foram agrupados e seguiram-se os passos de análise estatística segundo as variáveis categoria profissional e tempo de formação profissional.

Para verificar a existência de associação estatística entre a permissão da permanência da família na sala de emergência, a categoria profissional, o tempo de formação profissional, além de quem deve decidir se a família pode estar presente no atendimento, foi usado o teste qui-quadrado ou o teste exato de Fisher.^8^ No que se refere à relação entre a concordância com a presença da família na sala de emergência e o tipo de procedimento, aplicaram-se equações de estimação generalizada com função de distribuição binomial e ligação logito.^9^Todos os testes foram feitos com nível de significância de 5% e foi usado o SPSS15.0 (IBM, Chicago, Estados Unidos).

A análise das respostas oferecidas à questão qualitativa "o que você pensa sobre a família permanecer no atendimento à criança na sala de emergência?" seguiu os passos da codificação temática.^10^ Os dados inicialmente foram interpretados individualmente e foram desenvolvidos códigos que fossem adequados à opinião de cada participante. Posteriormente, os dados foram agrupados por suas similaridades, entre motivos pelos quais os profissionais incluem e excluem as famílias da sala de emergência.

O estudo foi aprovado pelos Comitês de Ética do HU-USP (protocolo 1182/12) e da Escola de Enfermagem da USP (protocolo 1102/2011). O período de coleta de dados foi em março de 2012.

## Resultados

Foram convidados a participar do estudo todos os profissionais de saúde da unidade (n=46) e a taxa de devolução dos questionários foi de 100%. A maioria dos profissionais era do sexo feminino (78,3%), pertencente à equipe de enfermagem (56,5%), relatou já ter participado de um atendimento de emergência em que a família estivesse presente (95,7%) e com 10 anos ou mais de formação profissional (65,2%). Fazendo a distribuição entre as categorias profissionais e o tempo de formação profissional: 70% da equipe de enfermagem tinham 10 anos ou mais de formação, enquanto o mesmo ocorria com apenas 30% da equipe médica.

Como praticamente toda a amostra já havia tido a experiência de um atendimento com a família dentro da sala de emergência, não foi feita a associação estatística entre essa variável e a opinião dos profissionais em relação à prática analisada.

De acordo com a Tabela 1, a opinião dos profissionais em relação à presença da família em sala de emergência não se associou à categoria profissional para nenhum dos procedimentos estudados. Os dados sugerem, porém, que a equipe médica foi mais favorável à presença da família durante todos os procedimentos invasivos estudados, quando comparada com a equipe de enfermagem.

**Tabela 1:** Opinião dos profissionais de saúde em relação à presença da família em sala de emergência pediátrica, segundo categoria profissional e tipo de procedimento

Procedimento Invasivo/Favorável à presença da família?	Equipe médica	Equipe de enfermagem	Total	*p*-valor
	n (%)	n (%)	n (%)	
*Punção venosa periférica*				
Sim	20 (100,0)	26 (100,0)	46 (100,0)	#

*Coleta de líquor*				
Sim	19 (95,0)	23 (88,5)	42 (91,3)	0,622
Não	1 (5,0)	3 (11,5)	4 (8,7)	

*Punção intraóssea*				
Sim	15 (75,0)	15 (57,7)	30 (65,2)	0,222
Não	5 (25,0)	11 (42,3)	16 (34,8)	

*Intubação traqueal*				
Sim	14 (70,0)	11 (42,3)	25 (54,3)	0,062
Não	6 (30,0)	15 (57,7)	21 (45,7)	

*RCP*				
Sim	13 (65,0)	11 (42,3)	24 (52,2)	0,127
Não	7 (35,0)	15 (57,7)	22 (47,8)	
*Total*	20 (100,0)	26 (100,0)	46 (100,0)	

RCP, reanimação cardiopulmonar.

# Todos os profissionais aceitam a presença da família para esse procedimento.

Na Tabela 2, observa-se que os profissionais com menos tempo de formação profissional consideraram em maior proporção do que aqueles com 10 anos ou mais de formados que a família pode estar presente ns procedimentos de intubação traqueal e RCP (*p*=0,007 e *p*=0,024, respectivamente). Para os demais procedimentos, os dados também indicam, embora sem associação estatística, que os profissionais formados há menos tempo são mais favoráveis à permanência da família na sala de emergência.

**Tabela 2:** Opinião dos profissionais de saúde em relação à presença da família em sala de emergência pediátrica, segundo tempo de formação profissional e tipo de procedimento

Procedimento Invasivo/ Favorável à presença da família?	Tempo de formação profissional		Total	*p*-valor
	<10 anos n (%)	≥10 anos n (%)	n (%)	
*Punção venosa periférica*				
Sim	16 (100,0)	30 (100,0)	46 (100,0)	a

*Coleta de líquor*				
Sim	16 (100,0)	26 (86,7)	42 (91,3)	0,282
Não	0 (0,0)	4 (13,3)	4 (8,7)	*

*Punção intraóssea*				
Sim	13 (81,3)	17 (56,7)	30 (65,2)	0,095
Não	3 (18,8)	13 (43,3)	16 (34,8)	

Intubação traqueal				
Sim	13 (81,3)	12 (40,0)	25 (54,3)	0,007
Não	3 (18,8)	18 (60,0)	21 (45,7)	

*RCP*				
Sim	12 (75,0)	12 (40,0)	24 (52,2)	**0,024**
Não	4 (25,0)	18 (60,0)	22 (47,8)	
*Total*	16 (100,0)	30 (100,0)	46 (100,0)	

RCP, reanimação cardiopulmonar.

a Todos os profissionais aceitam a presença da família para esse procedimento.

A maioria dos profissionais (69,8%) acredita que são os próprios profissionais de saúde que devem decidir se as famílias podem permanecer durante o atendimento na sala de emergência. Os profissionais com menos de 10 anos de formação profissional acreditam com maior frequência (*p*=0,034) que as famílias devem participar dessa decisão (Tabela 3).

**Tabela 3:** Opinião sobre quem deve decidir se a família pode ou não estar presente durante o atendimento em sala de emergência pediátrica, segundo categoria profissional e tempo de formação profissional

	Quem deve decidir se a família pode ou não permanecer na sala de emergência		
	Família n (%)	Profissionais de saúde n (%)	*p*-valor
*Categoria profissional*			0,707
Equipe médica	6 (33,3)	12 (66,7)	
Equipe de enfermagem	7 (28,0)	18 (72,0)	

*Tempo de formação profissional*			0,034
<10 anos	(53,3)	7 (46,7)	
≥10 anos	5 (17,9)	23 (82,1)	
*Total*	13 (30,2)	30 (69,8)	

Três casos foram excluídos dessa análise pela impossibilidade de determinar a resposta oferecida a essa questão.

Com respeito à relação entre a presença da família na sala de emergência pediátrica e a complexidade dos procedimentos, a seguinte ordem crescente de aceitação foi observada (Tabela 4): punção venosa periférica, coleta de líquor, punção intraóssea, intubação traqueal e RCP. A equipe de enfermagem se mostrou mais favorável à presença da família em procedimentos menos complexos (punção venosa periférica e coleta de líquor; *p*<0,001) em relação aos procedimentos mais complexos (punção intraóssea, intubação traqueal e RCP). Em contrapartida, a concordância da equipe médica em relação à presença da família foi similar (*p*=0,124), independentemente do procedimento.

**Tabela 4:** Opinião de cada categoria profissional em relação à presença da família em sala de emergência pediátrica de acordo com o procedimento

Categoria profissional	Procedimento invasivo	Favorável à presença da família em sala de emergência?		Total	*p*-valor
		Não n (%)	Sim n (%)		
Equipe de enfermagem	Punção venosa periférica	0 (0,0)	26 (100,0)	26	<0,001
	Coleta de líquor	3 (11,5)	23 (88,5)	26	
	Punção intraóssea	11 (42,3)	15 (57,7)	26	
	Intubação traqueal	15 (57,7)	11 (42,3)	26	
	RCP	15 (57,7)	11 (42,3)	26	
Equipe médica	Punção venosa periférica	0 (0,0)	20 (100,0)	20	0,124
	Coleta de líquor	1 (5,0)	19 (95,0)	20	
	Punção intraóssea	5 (25,0)	15 (75,0)	20	
	Intubação traqueal	6 (30,0)	14 (70,0)	20	
	RCP	7 (35,0)	13 (65,0)	20	
Total	Punção venosa periférica	0 (0,0)	46 (100,0)	46	<0,001
	Coleta de líquor	4 (8,7)	42 (91,3)	46	
	Punção intraóssea	16 (34,8)	30 (65,2)	46	
	Intubação traqueal	21 (45,7)	25 (54,3)	46	
	RCP	22 (47,8)	24 (52,2)	46	

Os motivos pelos quais os profissionais incluem as famílias na sala de emergência foram: a família observar os esforços para salvar a vida da criança, a família fornecer informações importantes sobre o paciente, ser um direito da família e a família proporcionar segurança à criança. Já os motivos que os levam a excluir as famílias foram: os profissionais não têm tempo para dar atenção às famílias, a família restringe o ensino a estudantes, a família interfere na atuação dos profissionais, a necessidade de um profissional permanecer junto com a família e a família permanecer com memórias negativas do atendimento.

## Discussão

Este estudo é pioneiro na literatura nacional e permite conhecer a opinião de profissionais de saúde atuantes em um pronto-socorro infantil em relação à presença da família em sala de emergência. Como geralmente são os profissionais de saúde que decidem se a família poderá estar presente no atendimento de emergência, conhecer suas opiniões é fundamental para que sejam traçadas estratégias que modifiquem o modo como a equipe de saúde cuida se aproxima e reconhece as necessidades das famílias no ambiente de emergência.

O número de profissionais que relataram já ter participado de um atendimento de emergência em que a família esteve presente é bem superior ao descrito na literatura - 23,1% a 70,1%.^11^^,^ 12 Esse dado pode significar um cenário positivo para a implantação de estratégias do modelo assistencial do CCF, pois a experiência prévia com a presença da família em situações de emergência mostra-se como o determinante mais favorável para essa prática.^13^

Apesar de a equipe médica ter se mostrado mais favorável à presença da família na sala de emergência do que a equipe de enfermagem, esse resultado se contrapõe aos descritos na literatura.^14^^,^ 15 Vale ressaltar que os estudos descritos na literatura internacional não incluem as perspectivas de auxiliares e técnicos de enfermagem.

O fato de os profissionais com menor tempo de formação profissional serem mais favoráveis à presença da família durante o atendimento de emergência reforça os resultados observados referentes ao fato de que a equipe médica foi mais favorável à presença da família em sala de emergência, uma vez que a maioria desses profissionais (70%) é formada há menos de 10 anos. Não há correlação na literatura entre tempo de experiência em serviço de emergência e atitudes tomadas pelos profissionais em relação à presença da família em sala de emergência.^16^

A maioria dos profissionais acredita que a família não deva participar da decisão sobre sua própria permanência na sala de emergência. Esses dados refletem principalmente a opinião dos profissionais formados há mais tempo. Resultados disponíveis na literatura também mostram que os profissionais de saúde desconsideram a autonomia da família nesse processo de tomada de decisão.^12^^,^ 17

A feitura mais frequente dos procedimentos menos invasivos no cotidiano parece conferir aos profissionais uma maior segurança nesses procedimentos, o que justificaria os achados na literatura.^17^^,^ 18 Eles mostram que quanto mais invasivo o procedimento, menos os profissionais são favoráveis à presença da família. Entretanto, em nosso estudo, somente a equipe de enfermagem demonstrou essa preferência em relação à presença da família apenas para os procedimentos menos invasivos.

Os motivos que levaram os profissionais a incluírem e excluírem as famílias da sala de emergência foram similares aos apresentados por médicos e enfermeiros em estudos internacionais. Isso demonstra que o contexto cultural pouco teve impacto na percepção da equipe de saúde ao decidir deixar ou não a família estar presente no atendimento.^19^^-^ 21 É fundamental que as necessidades das famílias, mesmo nas situações de emergência, sejam consideradas no cuidado prestado, pois a presença da família em uma situação na qual os membros da equipe de saúde não estão preparados para reconhecer suas necessidades pode ter consequências mais negativas para as famílias do que não estar ao lado do seu parente.^22^ Estudos mostram que, quando é dada a opção, as família em geral escolhem permanecer durante o atendimento de emergência e sua presença auxilia o processo de luto em casos de óbitos, propicia uma comunicação mais efetiva com a equipe multiprofissional e permite constatar que todos os esforças necessários foram feitos para salvar a vida do paciente.^23^^,^ 24

Um contexto favorável para promover a presença da família em sala de emergência inclui a elaboração de um programa de sensibilização dos profissionais para que a equipe se capacite para incluir as famílias nesse cenário e reconhecer os benefícios que a família confere ao atendimento de emergência.^25^ É necessária a criação de estratégias que permitam que as famílias estejam presentes na sala de emergência e considere suas necessidades. Isso pode ser alcançado ao se designar um membro da equipe de saúde para ficar exclusivamente com a família, proporcionar apoio às suas necessidades psicossociais, o que poderá colaborar para a ausência de memórias estressantes no futuro. Além disso, esse profissional - que pode ser qualquer membro da equipe multiprofissional - deve avaliar as respostas emocionais das famílias diante os procedimentos e prever possíveis interrupções no atendimento.^26^ Porém, para criar esse contexto favorável em nossa realidade cultural, primeiramente é necessário reconhecer que o contexto de emergência pediátrico brasileiro difere do descrito no cenário internacional e é marcado por sobrecarga de trabalho dos profissionais de saúde, decorrente da superlotação dos prontos-socorros e limitação de recursos humanos, físicos e materiais disponíveis. Além disso, o acesso irrestrito aos serviços de emergência resulta em longo de tempo de espera de atendimento para os usuários, o que ocasiona desgaste e estresse à equipe de saúde, que trabalha diante uma demanda de trabalho superior à capacidade que está preparada para atender.^27^^,^ 28

Este estudo tem como limitação expor a percepção de profissionais em uma determinada realidade e contexto e não poder ser reproduzido integralmente para outros cenários de emergência. Entretanto, configura-se em uma excelente ferramenta para que futuros estudos estabeleçam estratégias para incluir as famílias na sala de emergência. Ainda são necessários estudos nacionais que avaliem as percepções das famílias nesse contexto e a criação de um protocolo que normatize essa prática assistencial, adaptada para nossa realidade assistencial e cultural.

Por fim, o estudo conclui que a equipe médica e os profissionais com menos de 10 anos de formação profissional são mais favoráveis à presença da família em sala de emergência do que os demais profissionais. Os motivos determinantes para que os profissionais excluam as famílias da sala de emergência são relacionados ao desconforto e à insegurança sentidos pelos profissionais ao ter a família ao seu lado durante o atendimento. Já os motivos que os fazem incluir as famílias nesse cenário são ligados, muitas vezes, ao objetivo de facilitar o trabalho dos próprios profissionais e desconsideram as reais necessidades das famílias.
